# Developmental differences in the intestinal microbiota of Chinese 1-year-old infants and 4-year-old children

**DOI:** 10.1038/s41598-020-76591-4

**Published:** 2020-11-10

**Authors:** Min Guo, Maohua Miao, Yuezhu Wang, Mengmeng Duan, Fen Yang, Yao Chen, Wei Yuan, Huajun Zheng

**Affiliations:** 1 NHC Key Laboratory of Reproduction Regulation (Shanghai Institute of Planned Parenthood Research), Fudan University, Shanghai, 200032 China; 2 Shanghai-MOST Key Laboratory of Health and Disease Genomics, Chinese National Human Genome Center at Shanghai, Shanghai, 201203 China

**Keywords:** RNA sequencing, Microbiome

## Abstract

The microbiota profile of children changes with age. To investigate the differences in the gut microbiota profile of 1- and 4-year-old children, we collected fecal samples and sequenced the V3–V4 hypervariable region of the 16S rRNA gene via high-throughput DNA sequencing. From phylum to species level, the microbiota underwent significant changes with age. The abundance of phyla *Proteobacteria* and *Actinobacteria* declined with age, whereas phyla *Firmicutes* and *Bacteroidetes* increased with age and dominated the gut microbiota of 4-year-olds. The intestinal environment of children at age four is closer to maturity. Hence, the abundance of *Bifidobacterium* significantly decreased in the gut of 4-year-olds, whereas *Akkermansia muciniphila* increased from 0.14% in 1-year-olds to 4.25% in 4-year-olds. The functional change in gut microbiota is consistent with changes in infant food, as microbiota participating in amino acid and vitamin metabolism were enriched in 1-year-olds, whereas microbiota involved in lipid metabolism increased with age.

## Introduction

Intestinal microbiota play an important role in human health, and dysbiosis (an imbalance in microbiota) could cause immunologic dysregulation^1^ and various chronic diseases, including diabetes^2^, obesity^3^, inflammatory bowel disease^4^, rheumatoid arthritis^5^, autism spectrum disorders^6^, and cancers^7^. The gut–brain axis^8^, gut–liver axis^9^ and gut–lung axis^10^ imply a bidirectional interaction between microbiota and tissues of human body, emphasizing the role of microbiota in both health and disease.

Strikingly, intestinal microbiota imbalance early in life may lead to disease conditions at a later age^11,12^. The gut microbiota taxa of 2-year-old infants showed an increasingly strong association with the BMI of 12-year-old children^13^, and reduced bacterial diversity in both 1- and 12-month-old infants’ intestinal microbiota was associated with an increased risk of allergic sensitization in the first 6 years of life^14^. These findings drew more attention on the development of pediatric intestinal microbiota^15^.

The formation of infant gastrointestinal (GI) microbiota is affected by many factors, including mode of delivery^16,17^, type of feeding^18–20^, race and cord blood vitamin D levels^21^. Most studies support the opinion that vaginal delivery and breast-feeding contribute to healthy gut microbiota^22,23^, but growing infants displayed increased alpha-diversity and reduced beta-diversity in the gut microbiota^22^. Analyses have shown that infants possess individually distinct microbial profiles by the end of the first year of life, but begin converging towards the characteristic microbiota of adults^24^, and by age three, the infant microbiota gradually changed towards an adult-like structure^25^. New opinion suggested that infant microbiota development may take longer than 3 years, but the gut microbiota composition in childhood beyond age three is often overlooked^26^.

To fill the knowledge gap about gut microbiota beyond age three, we collected the stool samples of 1- and 4-year-old healthy Chinese children and investigated whether they displayed a distinct microbial profile at age one and adult-like mature profile at age four.

## Materials and methods

### Sample collection

Study subjects were selected from the Shanghai-Minhang Birth Cohort Study (S-MBCS), which was reviewed and approved by the ethics committee board of the Shanghai Institute of Planned Parenthood Research (IRB00008297). Written informed consents were obtained from the parents of all participants involved in this study. All methods were performed in accordance with the Declaration of Helsinki. Fecal samples were collected from 1-year-old infants and 4-year-olds who were healthy, not overweight, had not taken antibiotics in one month, and had not developed eczema. All samples were stored at − 80 °C before DNA extraction.

### DNA extraction, PCR amplification and 16S rRNA gene sequencing

DNA extraction and PCR amplification were performed as described previously^27^, with some modifications. In brief, the genomic DNA was extracted from 300 mg of feces using a QIAamp DNA stool mini kit (Qiagen, Hilden, Germany) according to the manufacturer’s instructions. The integrity of extracted genomic DNA was checked by 1.5% agarose gel electrophoresis. To generate 16S rRNA gene amplicons, the V3-4 hypervariable region of the 16S rRNA genes was amplified using the primers 338F (5′-CCTACGGGNGGCWGCAG-3′) and 806R (5′-GACTACHVGGGTATCTAATCC-3′) with a TransStart Fastpfu DNA Polymerase (TransGen, Beijing, China) in 20 cycles. All amplicons were purified using the QIAquick PCR Purification Kit (Qiagen), quantified on Qubit (Life Technologies), then pooled into equal concentrations. The pooled amplicons were ligated with adaptors using TruePrep DNA Library Prep Kit V2 for Illumina (Vazyme,China), then 2 × 300 bp paired-end sequencing was performed on an Illumina MiSeq instrument with MiSeq Reagent Kit v3.

### Bioinformatics and statistical analysis

Paired-end 16S rRNA sequences were assembled using Mothur (version 1.41.1)^28^. DNA sequences were discarded using the following criteria: containing ambiguous bases, or containing chimeric or contaminant sequences, or homopolymers of > 8 nucleotides, or with lengths shorter than 350 bp. The chimeric sequences were identified by VSEARCH algorithm, and non-16S contaminants sequences were filtered based on the RDP database. Using SILVA reference databases (V132)^29^, the DNA sequences were clustered into OTUs at 97% similarity with reads number normalizing to 17,436. Community richness, evenness, and diversity (Shannon, Shannoneven, ACE, Chao, and Good’s coverage) were also assessed using Mothur. The online software, Ribosomal Database Project (RDP) classifier^30^ was used for taxonomy assignment for each Operational Taxonomic Units (OTU)^31^ using default parameters. Differences in bacterial diversity were assessed using analysis of similarities (ANOSIM), based on the unweighted UniFrac distance metrics using Mothur. The differences in features (taxonomy and OTU) were determined using STAMP tool via the Benjamini–Hochberg FDR test^32^. The prediction of microbiome functions were analyzed using Phylogenetic Investigation of Communities by Reconstruction of Unobserved States (PICRUSt) software^33^, based on Kyoto Encyclopedia of Genes and Genomes (KEGG) pathways^34^.

Representative OTUs were identified as species against the SILVA SSU database (132) and the NCBI online database with > 99% identity and highest total score^29,35^.

## Results

### Gut bacterial populations in 1- and 4-year-old children

A total of 10,210,871 (17,436–395,495) high-quality reads were obtained by high-throughput sequencing of 16S rRNA genes from 153 fecal samples (40 and 113 from 1- and 4-year-olds, respectively). To normalize data and avoid statistical bias, 17,436 16S rRNA genes of each sample were selected to calculate bacterial species richness, evenness, and diversity at 97% similarity. A total of 7195 OTUs (371 OTUs per sample on average) were obtained, and the Good’s coverage was over 99.8% for both groups (1- and 4-year-olds, Table 1), indicating that the sequencing depth was sufficient for gut microbiota investigation in children of two different ages.

**Table 1:** The gut microbiota diversity evaluation of 1-year and 4-years old children.

Group	Sample	OTUs	Coverage	Richness		Evenness	Diversity
				ACE	Chao	Shannoneven	Shannon
1-year	40	1159	0.998975	1580.63	1584.31	0.566114	3.99411
4-years	113	7089	0.999931	7103.50	7106.58	0.56353	4.99643

### Bacterial composition changes with age

**Figure 1:**
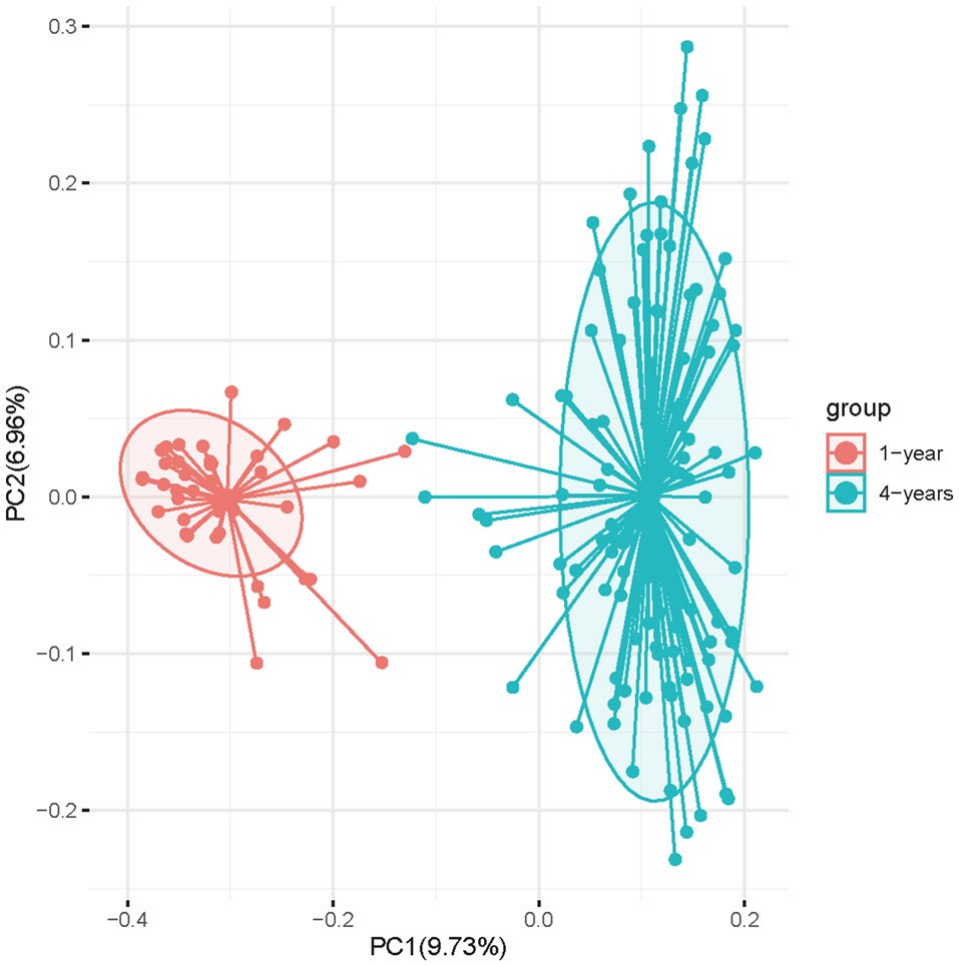
Principal coordinates analysis (PCoA) plots based on unweighted UniFrac distance metrics. Red points and blue points represent 1-year-old children and 4-year-old children, respectively.

**Figure 2:**
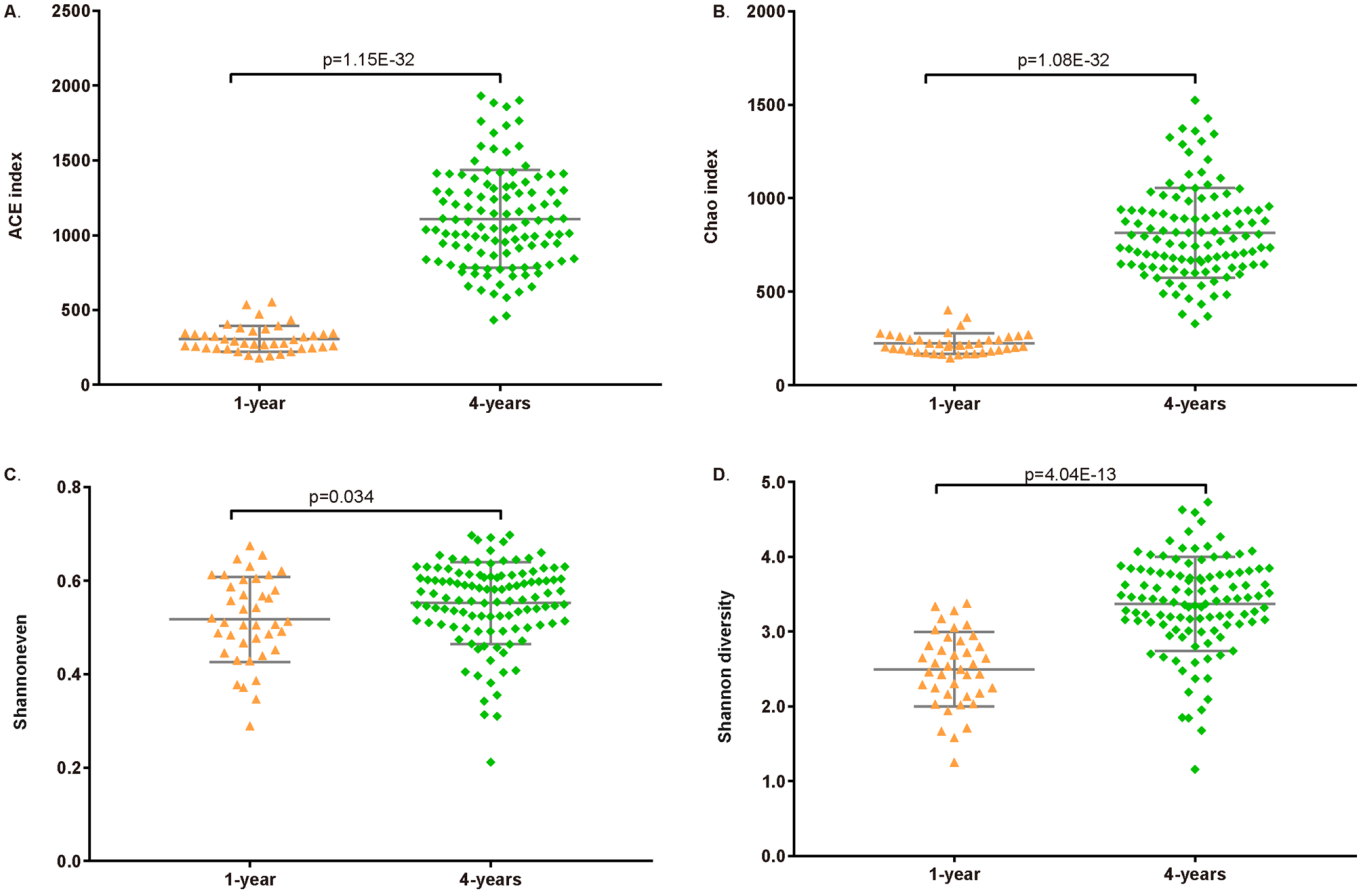
Richness, evenness and diversity of children gut microbiota. (**A**, **B**) were richness index, (**C**) was evenness index, (**D**) was diversity index. The p-value of top illustration was calculated by T-test, which indicated significant difference of gut microbiota between 1-year-old and 4-year-old children.

Based on the unweighted UniFrac distance metrics, principal coordinate analyses (PCoA) showed two significant parts divided by two different point of age (Fig. 1). ANOSIM analysis suggested that the microbial composition was significantly different (p < 0.001, R = 0.731) between 1- and 4-year-olds. Bacterial population evaluation showed that the species richness (ACE and Chao), species evenness, and diversity were significantly lower in the gut of 1-year-olds than in that of 4-year-olds (Fig. 2, p < 0.05). These results indicate that microbiota composition and diversity increased with the age of children.

**Figure 3:**
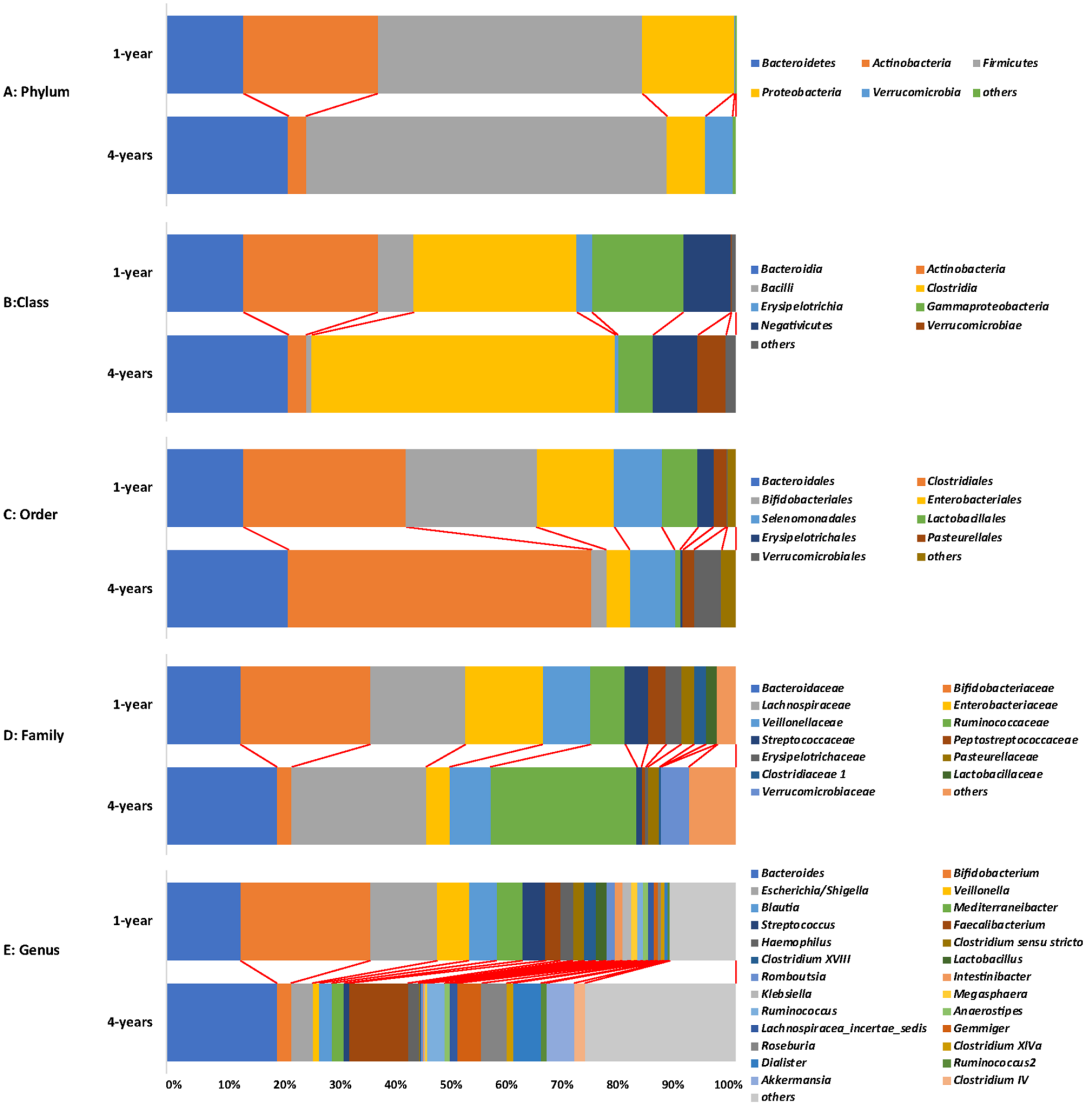
The gut microbiota composition of 1-year-old and 4-years-old children on different taxa level. The main bacteria phylum, class, order, family and genus in children gut were illustrated in different colors.

**Figure 4:**
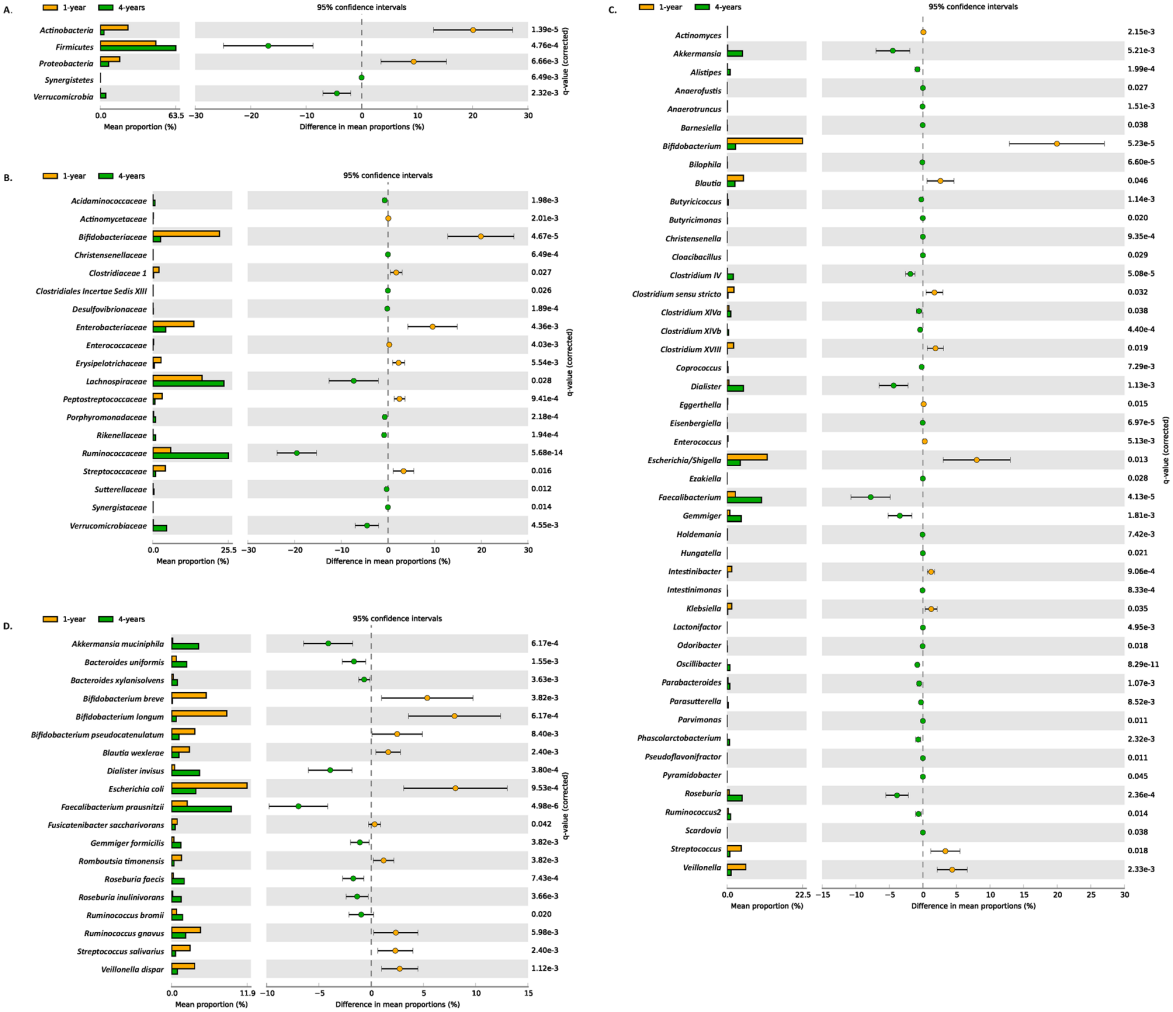
Gut microbiota comparison between 1-year and 4-yearold children at phylum level (**A**), family level (**B**), genus level (**C**) and species level (**D**). The taxa with significant difference (q-value < 0.05, computed by STAMP) between the two groups are shown.

From phylum to species level, the microbiota differed significantly between 1- and 4-year-olds. At the phylum level, a total of 12 phyla were confirmed, with five major phyla (*Actinobacteria*, *Bacteroidetes*, *Firmicutes*, *Proteobacteria*, and *Verrucomicrobia*) composing > 99% of gut microbiota (Fig. 3), and three phyla unique to 4-year-olds. Phyla *Actinobacteria* and *Proteobacteria* showed significant enrichment in 1-year-olds (q < 0.05), while phyla *Firmicutes*, *Synergistetes*, and *Verrucomicrobia* were significantly enriched in 4-year-olds (Fig. 4A).

At the class level, a total of 20 classes were revealed; 16 classes were identified in both 1- and 4-year-olds and nine classes were significantly different between both groups (Figure S1), with *Actinobacteria*, *Bacilli*, *Erysipelotrichia* and *Gammaproteobacteria* significantly enriched in 1-year-olds, and *Betaproteobacteria*, *Clostridia*, *Deltaproteobacteria*, *Synergistia* and *Verrucomicrobiae* significantly increased in 4-year-olds.

At the order level, 38 orders were identified in total; 28 orders were identified in both groups, and nine orders were significantly different between the groups (Figure S2), including *Bifidobacteriales*, *Enterobacteriales*, *Erysipelotrichales* and *Lactobacillales* enriched in 1-year-olds and *Burkholderiales*, *Clostridiales*, *Desulfovibrionales*, *Synergistales*, and *Verrucomicrobiales* enriched in 4-year-olds.

At the family level, 81 families were identified in total; 58 families were identified in both groups, 19 families were significantly different between the groups (Fig. 4B). Eight families, *Actinomycetaceae, Bifidobacteriaceae*, *Clostridiaceae 1*, *Enterobacteriaceae*, *Enterococcaceae, Erysipelotrichaceae*, *Peptostreptococcaceae* and *Streptococcaceae* were significantly enriched in 1-year-olds, while the other 11 families, like *Lachnospiraceae*, *Ruminococcaceae* and *Verrucomicrobiaceae* were significantly enriched in 4-year-olds.

At the genus level, 203 genera were identified in total; 133 genera were identified in both groups, 18 of which were major genera in both groups (> 1% per group Table 2). The fecal microbiome of 1-year-olds was generally dominated by *Bifidobacterium*, *Escherichia/Shigella*, and *Bacteroides*, each genus composing > 12% (total 47.4%) of the microbiome. While in 4-year-olds, the dominant genera were *Bacteroides* (19.3%) and *Faecalibacterium* (10.2%). A total of 46 genera (including 11 major genera) were significantly different between both groups (Fig. 4C). Noteworthily, 12 genera including *Actinomyces*, *Blautia*, *Clostridium *sensu stricto, *Clostridium XVIII*, *Eggerthella*, *Intestinibacter*, *Klebsiella*, *Streptococcus*, *Bifidobacterium*, *Escherichia/Shigella* and *Veillonella*) were significantly enriched in 1-year-olds. The other 34 genera, including *Akkermasia*, *Dialister*, *Faecalibacterium*, *Gemmiger*, *Roseburia*, etc., were significantly enriched in 4-year-olds. The dominant genus, *Bacteroides*, maintained a stable population with age (12.9% and 19.3% in 1- and 4-year-olds, respectively).

**Table 2:** Dominant genera and significant difference between 1-year and 4-years old children.

Genus	Feature	1-year: mean rel. freq. (%)	1-year: std. dev. (%)	4-years: mean rel. freq. (%)	4-years: std. dev. (%)	q-values	Enriched in
*Actinomyces*	Difference	0.1017	0.1187	0.0239	0.0296	0.0021	1-year
*Akkermansia*	Major and difference	0.1368	0.7098	4.6110	13.4165	0.0052	4-years
*Alistipes*	Difference	0.0612	0.2742	0.8799	1.8020	0.0002	4-years
*Anaerofustis*	Difference	0.0006	0.0035	0.0034	0.0091	0.0268	4-years
*Anaerotruncus*	Difference	0.0194	0.0529	0.0750	0.1206	0.0015	4-years
*Barnesiella*	Difference	0.0000	0.0000	0.0307	0.1228	0.0376	4-years
*Bifidobacterium*	Major and difference	22.4657	21.6951	2.4992	4.4353	0.0001	1-year
*Bilophila*	Difference	0.0016	0.0061	0.0827	0.1719	0.0001	4-years
*Blautia*	Major and difference	4.8976	5.9816	2.2828	2.9867	0.0458	1-year
*Butyricicoccus*	Difference	0.0978	0.2197	0.3386	0.5161	0.0011	4-years
*Butyricimonas*	Difference	0.0003	0.0017	0.0185	0.0657	0.0202	4-years
*Christensenella*	Difference	0.0000	0.0000	0.0070	0.0177	0.0009	4-years
*Cloacibacillus*	Difference	0.0000	0.0000	0.0017	0.0063	0.0293	4-years
*Clostridium IV*	Major and difference	0.0136	0.0587	1.8773	3.7870	0.0001	4-years
*Clostridium *sensu stricto	Major and difference	1.9968	3.8008	0.2728	0.6705	0.0324	4-years
*Clostridium XlVa*	Major and difference	0.5308	0.7931	1.1052	1.8900	0.0381	4-years
*Clostridium XlVb*	Difference	0.0067	0.0195	0.4134	0.9732	0.0004	4-years
*Clostridium XVIII*	Major and difference	1.9694	3.7634	0.0981	0.1562	0.0186	1-year
*Coprococcus*	Difference	0.0324	0.1297	0.2113	0.5193	0.0073	4-years
*Dialister*	Major and difference	0.5084	2.8026	4.8636	10.4218	0.0011	4-years
*Eggerthella*	Difference	0.1535	0.2235	0.0381	0.0516	0.0151	1-year
*Eisenbergiella*	Difference	0.0000	0.0000	0.0294	0.0620	0.0001	4-years
*Enterococcus*	Difference	0.2863	0.4723	0.0060	0.0254	0.0051	1-year
*Escherichia/Shigella*	Major and difference	11.9535	14.2319	3.9337	10.4966	0.0126	1-year
*Ezakiella*	Difference	0.0008	0.0038	0.0105	0.0362	0.0284	4-years
*Faecalibacterium*	Major and difference	2.4513	4.7935	10.2261	13.3559	0.0000	4-years
*Gemmiger*	Major and difference	0.7854	2.5049	4.2201	8.4445	0.0018	4-years
*Holdemania*	Difference	0.0177	0.0735	0.0756	0.1338	0.0074	4-years
*Hungatella*	Difference	0.0011	0.0040	0.0110	0.0356	0.0214	4-years
*Intestinibacter*	Major and difference	1.4063	1.6740	0.1925	0.3323	0.0009	1-year
*Intestinimonas*	Difference	0.0000	0.0000	0.0559	0.1416	0.0008	4-years
*Klebsiella*	Major and difference	1.3742	2.7721	0.1372	0.4604	0.0355	1-year
*Lactonifactor*	Difference	0.0008	0.0037	0.0051	0.0116	0.0050	4-years
*Odoribacter*	Difference	0.0000	0.0000	0.0418	0.1476	0.0182	4-years
*Oscillibacter*	Difference	0.0125	0.0326	0.8457	1.0766	0.0000	4-years
*Parabacteroides*	Difference	0.2177	0.5702	0.7776	1.0870	0.0011	4-years
*Parasutterella*	Difference	0.0118	0.0562	0.2985	0.9139	0.0085	4-years
*Parvimonas*	Difference	0.0011	0.0032	0.0058	0.0148	0.0112	4-years
*Phascolarctobacterium*	Difference	0.0089	0.0474	0.7095	1.9686	0.0023	4-years
*Pseudoflavonifractor*	Difference	0.0000	0.0000	0.0011	0.0038	0.0111	4-years
*Pyramidobacter*	Difference	0.0003	0.0017	0.0062	0.0245	0.0454	4-years
*Roseburia*	Major and difference	0.5940	1.6508	4.4477	8.4708	0.0002	4-years
*Ruminococcus2*	Major and difference	0.3787	0.9090	1.0312	1.6200	0.0144	4-years
*Scardovia*	Difference	0.0000	0.0000	0.0010	0.0040	0.0379	4-years
*Streptococcus*	Major and difference	4.1816	6.7029	0.8335	1.3996	0.0182	1-year
*Bacteroides*	Major	12.9731	19.2120	19.29113	19.1440	0.2184	4-year
*Veillonella*	Major and difference	5.5698	6.5315	1.2074	3.7846	0.0023	1-year

At the species (OTU) level, 23 of the top 30 OTUs were significantly different between 1- and 4-year-olds (Table S1), with 19 of them confirmed as known species via NCBI BLAST (Fig. 4D). Ten species were significantly enriched in 1-year-olds, including *Bifidobacterium breve*, *Bifidobacterium longum*, *Bifidobacterium pseudocatenulatum*, *Blautia wexlerae*, *Fusicatenibacter saccharivorans*, *Romboutsia timonensis*, *Ruminococcus gnavus*, *Streptococcus salivarius*, *Escherichia coli*, and *Veillonella dispar*, which occupied 39.9% of gut microbiota in 1-year-olds. The abundance of the other nine species, including *Akkermansia muciniphila*, *Bacteroides uniformis*, *Bacteroides xylanisolvens*, *Gemmiger formicilis*, *Roseburia faecis*, *Roseburia inulinivorans*, *Ruminococcus bromii*, *Faecalibacterium prausnitzii*, and *Dialister invisus*, increased significantly in 4-year-olds compared to that in 1-year-olds.

### Predicted functional change of gut microbiota between 1-year-old and 4-year-old children

We used PICRUSt software to predict the potential functional changes with age. Sixty-nine predicted Metabolism Pathways, 17 pathways related to Genetic Information Processing, two pathways belonging to Cellular Processes, and three pathways participating Environmental Information Processing, were identified as having significant differences (q-value < 0.05) between 1- and 4-year-olds (Figure S3). Analysis revealed the relative abundance of genes involved in O-glycan biosynthesis, steroid biosynthesis, secondary bile acid biosynthesis, carotenoid biosynthesis, etc., were significantly increased in 4-year-olds. Genes participating in cell motility, membrane transport, galactose metabolism, folate biosynthesis, glutathione metabolism, etc., were significantly decreased in 4-year-olds.

## Discussion

In this study, we compared the gut microbiota of 1- and 4-year-old Chinese children by investigating the V3–V4 hypervariable region of the 16S rRNA gene via high-throughput DNA sequencing. Our results revealed that the gut microbiota in children increased significantly from age 1 to 4. In terms of population, species richness, species evenness, and diversity were mainly dominated by five phyla (*Actinobacteria*, *Proteobacteria*, *Firmicutes*, *Bacteroidetes* and *Verrucombicrobia*). These findings are consistent with previous reports that the gut microbiome of children gradually mature as that of adults during the first three years of life^25^.

A lot of research in the past decades have focused on the development of the infant gut microbiota during the first 3 years of life; only, few reports have investigated the variation in gut microbiota in children above 3 years of age^26^, and further studies are needed. For example, Fiona et al*.* indicated that the gut microbiota of children was dynamic before age four due to the effects of perinatal factors^36^, and Ringel-Kulka et al*.* revealed that by age four the microbiota of children were still not as mature as those of adults, suggesting that the microbiota of children continue to progress after age 4^37^.

Hence, our research paid attention to the development of children’s gut microbiota at age four for a deeper understanding of the intestinal microbiota of children. Through the unweighted UniFrac distance metrics, we demonstrated that there was a significant difference in gut microbiota compositions between 1- and 4-year-old children, suggesting that the gut microbiota of infants matures with age.

We analyzed specific differences from the phylum to the species level. At the phylum level, *Actinobacteria* and *Proteobacteria* were significantly reduced in the intestines of 4-year-olds. This result is consistent with previous reports that *Actinobacteria*, represented by *Bifidobacterium*, declined after weaning due to decreased protein requirements^22,38,39^. However, *Firmicutes*, *Synergistetes*, and *Verrucomicrobia* increased significantly in the intestines of 4-year-olds. It was recently noted that the abundance of *Firmicutes* is suppressed while children receive breast milk^20^. Once weaning begins, *Firmicutes* increase in abundance and dominate gut microbiota. It is supposed that the introduction of solid foods can increase bacterial load and short-chain fatty acid levels, which may be due to the ability of *Firmicutes,* such as *Roseburia* spp., to metabolize carbohydrates in the diet^40,41^. *Bacteroides,* which can breakdown complex plant polysaccharides^42^, maintain dominance of the gut microbiota with age, indicating that *Bacteroides* already attained stability at infancy. These results are consistent with previous reports that *Firmicutes* and *Bacteroidetes* are the most dominant phyla in healthy adult subjects^43^, suggesting a maturation of gut microbiota at age 4.

At the genus level, *Bifidobacterium*, *Escherichia/Shigella*, and *Veillonella* were enriched in the intestines of 1-year-olds. *Bifidobacterium* levels declined with age, in agreement with reports that *Bifidobacterium* is more abundant in children than in adults^44^. It is generally known that *Bifidobacterium* has several subspecies relating to infants. *Bifidobacterium longum* subsp. is a kind of archetypical bacteria capable of using human milk oligosaccharide (HMO) as substrates. *B. longum* subsp. *infantis* is an infant commensal that thrives in the presence of milk^45^. Our results showed that the abundance of *Bifidobacterium* decreased significantly in the gut microbiota of 4-year-olds, coinciding with the beginning of weaning and the introduction of table foods. In the gut microbiota of 4-year-olds, the abundance of *Faecalibacterium, Dialister, Gemmiger*, and *Akkermansia* increased significantly. Notably, *Akkermansia* is a probiotic, and may be more beneficial for growing children as they face a more complex diet and other environmental factors. *Akkermansia* helps regulate the thickness of intestinal mucus and maintain intestinal barrier integrity to reduce sugar absorption. It is beneficial for weight loss, blood sugar regulation and diabetes mellitus management^46^.

At the species level, *F. prausnitzii*, *D. invisus*, *A. muciniphila*, *R. bromii*, *B. xylanisolvens*, *G. formicilis*, *R. faecis* and *R. inulinivorans* increased significantly with age, while *E. coli*, *B. pseudocatenulatum, B. longum, B. wexlerae*, *F. saccharivorans*, *R. timonensis*, *R. gnavus*, *S. salivarius*, *V. dispar* and *B. breve* were more abundant in the intestines of 1-year-olds than in those of 4-year-olds. *F. prausnitzii* is one of the most abundant microorganisms in the intestinal tract of healthy people; it can generate butyrate as an anti-inflammatory to help slow down inflammatory bowel disease^46^. *A. muciniphila*, a mucin-degrading probiotic, increased from 0.14% in 1-year-olds to 4.25% in 4-year-olds. *A. muciniphila* can regulate immune responses by promoting relevant gene expression^47^, and reverse high-fat diet-induced metabolic disorders by improving host metabolism^46^. *R. inulinivorans* belong to the genus *Roseburia,* which was reported to produce short-chain fatty acids and play a major role in maintaining gut health and immune defense^48^. The increased levels of these profitable species suggest that the intestinal environment of 4-year-olds is attaining adult-like maturity.

*Bifidobacterium* is able to thrive on HMOs and is dominant in infant gut microbiota before weaning^24^. The abundance of these milk-related *Bifidobacterium*, including *B. pseudocatenulatum, B. longum*, and *B. breve*, significantly decreased in 4-year-olds due to the change in their diet (from milk to solid food). The onset of weaning is usually associated with an increase in a diversity of intestinal microbiota, with Bifidobacteria-dominated intestinal microbiota gradually being replaced by more complex microbial communities capable of degrading carbohydrates from plant and animal sources.

In this study, PICRUSt software was used to predict the potential function of the gut microbiota of 1- and 4-year-olds. Bacteria involved in galactose metabolism, amino acid metabolism, cofactor and vitamin metabolism like folate biosynthesis, nicotinate and nicotinamide metabolism, vitamin B6 metabolism, etc., were significantly enriched in 1-year-olds. While the abundance of microbiota participating in lipid metabolism, metabolism of terpenoids and polyketides pathways increased with age. This functional change in microbiota is consistent with children’s diet changes.

Nevertheless, the current study has limitations as this study used fecal samples. As is known, the fecal microbiota does not fully represent the luminal or mucosal communities of the GI tract^49,50^. Although previous study revealed that fecal microbial community has a good potential to identify most taxa in the chicken gut^51^, non-invasively sampling at different gut locations would be preferred, and recently developed smart sampling capsule would achieve this goal^52^. Another limitation of our study is lacking of paying attention to the factors influencing the development of intestinal microbiota in children, such as breast milk feeding, dietary habits and antibiotic use.

In conclusion, the first 4 years of life is a crucial period for the formation of intestinal microbiota in young children, and has a profound impact on subsequent physical development and health. Our study demonstrates that the intestinal microbiota composition of infants changed from Bifidobacteria-dominated to a more complex microbiota, and attained adult-like intestinal microbiota maturation by age four. It is worthy of note that owing to various influencing factors, there are great differences in the composition and development of intestinal microbiota among different populations; our study is scientifically relevant among existing studies involving other ethnic groups.

## Supplementary information

Supplementary Information.

## Data Availability

The microbiota sequence data for the 1- and 4-year-old children have been deposited in the National Omics Data Encyclopedia (NODE, https://www.biosino.org/node/index) under the accession numbers OEX010570 and OEX010571, respectively.

## Abbreviations

GI: Gastrointestinal
S-MBCS: Shanghai-Minhang Birth Cohort Study
RDP: Ribosomal Database Project
OTU: Operational Taxonomic Units
ANOSIM: Analysis of similarities
PICRUSt: Phylogenetic Investigation of Communities by Reconstruction of Unobserved States
KEGG: Kyoto Encyclopedia of Genes and Genomes
PCoA: Principal coordinate analyses
HMO: Human milk oligosaccharide
