# Supplementary material for: Developmental differences in the intestinal microbiota of Chinese 1-year-old infants and 4-year-old children
Source: Sci Rep. 2020 Nov 10;10:19470. doi: 10.1038/s41598-020-76591-4 (PMC7656257; doi:10.1038/s41598-020-76591-4)
Supplement: Supplementary file 1 — Supplementary Information. [file 41598_2020_76591_MOESM1_ESM.pdf]

Supplementary Information

Developmental differences in the Intestinal Microbiota of Chinese 1-year-old Infants and 4-year-old children

Min Guo[1]†, Maohua Miao[1]†, Yuezhu Wang[2], Mengmeng Duan[1], Fen Yang[1], Yao Chen[1], Wei Yuan[1]*, Huajun Zheng[1]*

1. NHC Key Laboratory of Reproduction Regulation (Shanghai Institute of Planned Parenthood Research), Fudan University, Shanghai, 200032, China.

2. Shanghai-MOST Key Laboratory of Health and Disease Genomics, Chinese National Human Genome Center at Shanghai, Shanghai, 201203, China.

[†]These authors contributed equally to this work

*corresponding author

Wei Yuan, yuanwei11@yahoo.com

Huajun Zheng, zhenghj@chgc.sh.cn

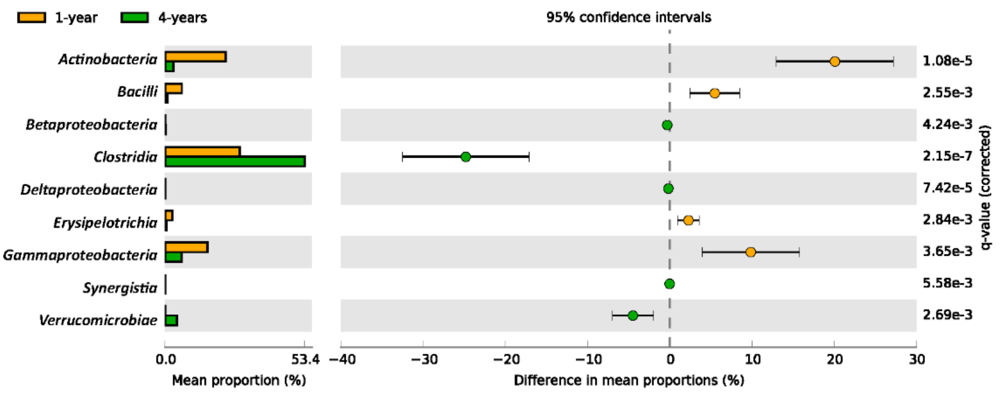

Figure S1. Significant difference of gut microbiota between 1-year and 4-years old children at class level.

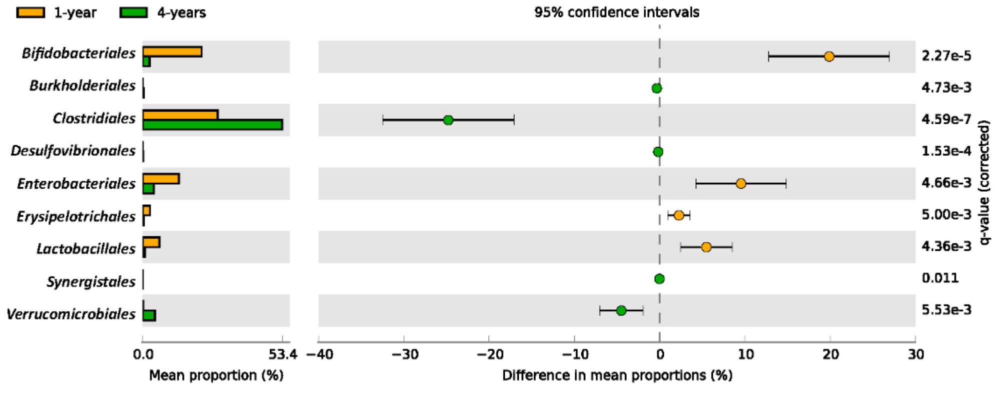

Figure S2. Significant difference of gut microbiota between 1-year and 4-years old children at order level.

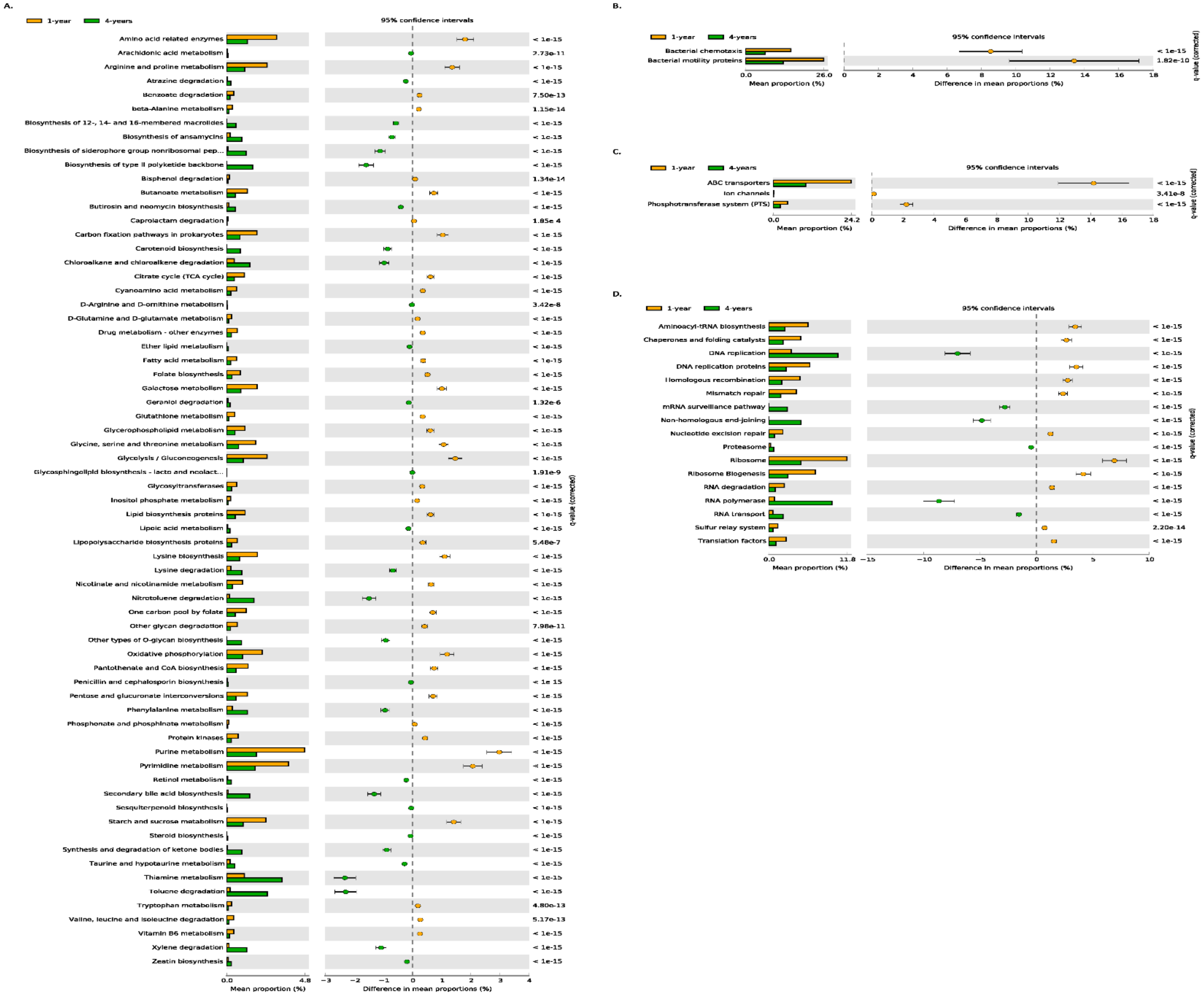

A.
1-year
4-years
95% confidence intervals
Amino acid related enzymes < 1e-15
Arachidonic acid metabolism 2.73e-11
Arginine and proline metabolism < 1e-15
Atrazine degradation < 1e-15
Benzoate degradation 7.50e-13
beta-Alanine metabolism 1.15e-14
Biosynthesis of 12-, 14- and 16-membered macrolides < 1e-15
Biosynthesis of ansamycins < 1e-15
Biosynthesis of siderophore group nonribosomal pep... < 1e-15
Biosynthesis of type II polyketide backbone < 1e-15
Bisphenol degradation 1.34e-14
Butanoate metabolism < 1e-15
Butirosin and neomycin biosynthesis < 1e-15
Caprolactam degradation 1.85e-4
Carbon fixation pathways in prokaryotes < 1e-15
Carotenoid biosynthesis < 1e-15
Chloroalkane and chloroalkene degradation < 1e-15
Citrate cycle (TCA cycle) < 1e-15
Cyanoamino acid metabolism < 1e-15
D-Arginine and D-ornithine metabolism 3.42e-8
D-Glutamine and D-glutamate metabolism < 1e-15
Drug metabolism - other enzymes < 1e-15
Ether lipid metabolism < 1e-15
Fatty acid metabolism < 1e-15
Folate biosynthesis < 1e-15
Galactose metabolism < 1e-15
Geraniol degradation 1.32e-6
Glutathione metabolism < 1e-15
Glycerophospholipid metabolism < 1e-15
Glycine, serine and threonine metabolism < 1e-15
Glycolysis / Gluconeogenesis < 1e-15
Glycosphingolipid biosynthesis - lacto and neolact... 1.91e-9
Glycosyltransferases < 1e-15
Inositol phosphate metabolism < 1e-15
Lipid biosynthesis proteins < 1e-15
Lipoic acid metabolism < 1e-15
Lipopolysaccharide biosynthesis proteins 5.48e-7
Lysine biosynthesis < 1e-15
Lysine degradation < 1e-15
Nicotinate and nicotinamide metabolism < 1e-15
Nitrotoluene degradation < 1e-15
One carbon pool by folate < 1e-15
Other glycan degradation 7.98e-11
Other types of O-glycan biosynthesis < 1e-15
Oxidative phosphorylation < 1e-15
Pantothenate and CoA biosynthesis < 1e-15
Penicillin and cephalosporin biosynthesis < 1e-15
Pentose and glucuronate interconversions < 1e-15
Phenylalanine metabolism < 1e-15
Phosphonate and phosphinate metabolism < 1e-15
Protein kinases < 1e-15
Purine metabolism < 1e-15
Pyrimidine metabolism < 1e-15
Retinol metabolism < 1e-15
Secondary bile acid biosynthesis < 1e-15
Sesquiterpenoid biosynthesis < 1e-15
Starch and sucrose metabolism < 1e-15
Steroid biosynthesis < 1e-15
Synthesis and degradation of ketone bodies < 1e-15
Taurine and hypotaurine metabolism < 1e-15
Thiamine metabolism < 1e-15
Toluene degradation < 1e-15
Tryptophan metabolism 4.80e-13
Valine, leucine and isoleucine degradation 5.17e-13
Vitamin B6 metabolism < 1e-15
Xylene degradation < 1e-15
Zeatin biosynthesis < 1e-15
0.0 4.8
Mean proportion (%)
−3 −2 −1 0 1 2 3 4
Difference in mean proportions (%)
q-value (corrected)
B.
1-year
4-years
95% confidence intervals
Bacterial chemotaxis < 1e-15
Bacterial motility proteins 1.82e-10
0.0 26.0
Mean proportion (%)
0 2 4 6 8 10 12 14 16 18
Difference in mean proportions (%)
q-value (corrected)
C.
1-year
4-years
95% confidence intervals
ABC transporters < 1e-15
Ion channels 3.41e-8
Phosphotransferase system (PTS) < 1e-15
0.0 24.2
Mean proportion (%)
0 2 4 6 8 10 12 14 16 18
Difference in mean proportions (%)
q-value (corrected)
D.
1-year
4-years
95% confidence intervals
Aminoacyl-tRNA biosynthesis < 1e-15
Chaperones and folding catalysts < 1e-15
DNA replication < 1e-15
DNA replication proteins < 1e-15
Homologous recombination < 1e-15
Mismatch repair < 1e-15
mRNA surveillance pathway < 1e-15
Non-homologous end-joining < 1e-15
Nucleotide excision repair < 1e-15
Proteasome < 1e-15
Ribosome < 1e-15
Ribosome Biogenesis < 1e-15
RNA degradation < 1e-15
RNA polymerase < 1e-15
RNA transport < 1e-15
Sulfur relay system 2.20e-14
Translation factors < 1e-15
0.0 11.8
Mean proportion (%)
−15 −10 −5 0 5 10
Difference in mean proportions (%)
q-value (corrected)

Figure S3: Significantly different pathways based on PICRUSt prediction. A: metabolism, B: Cellular Processes, C: Environmental Information Processing, D: Genetic Information Processing.

Table S1. Dominant species and significant differences between 1-year old and 4-years old children.

species	taxonomy	1-year: mean rel. freq. (%)	1-year: std. dev. (%)	4-years: mean rel. freq. (%)	4-years: std. dev. (%)	q-values	enriched in
Akkermansia muciniphila	*Verrucomicrobia;Verrucomicrobiae;Verrucomicrobiales; Verrucomicrobiaceae; Akkermansia*	0.1365	0.7081	4.2467	12.5290	0.0006	4-years
Bacteroides uniformis	*Bacteroidetes;Bacteroidia;Bacteroidales;Bacteroidaceae;Bacteroides*	0.7229	2.1046	2.3728	4.8382	0.0015	4-years
Bacteroides xylanisolvens	*Bacteroidetes;Bacteroidia;Bacteroidales;Bacteroidaceae;Bacteroides*	0.2455	0.7294	0.9195	2.5747	0.0036	4-years
Bifidobacterium breve	*Actinobacteria;Actinobacteria;Bifidobacteriales;Bifidobacteriaceae; Bifidobacterium*	5.4473	13.5277	0.0833	0.4174	0.0038	1-year
Bifidobacterium longum	*Actinobacteria;Actinobacteria;Bifidobacteriales;Bifidobacteriaceae; Bifidobacterium*	8.7024	13.6215	0.7241	1.8352	0.0006	1-year
Bifidobacterium pseudocatenulatum	*Actinobacteria;Actinobacteria;Bifidobacteriales;Bifidobacteriaceae; Bifidobacterium*	3.6506	7.2775	1.1687	2.8872	0.0084	1-year
Blautia wexlerae	*Firmicutes;Clostridia;Clostridiales;Lachnospiraceae;Blautia*	2.7893	3.5496	1.1586	1.6920	0.0024	1-year
Dialister invisus	*Firmicutes;Negativicutes;Selenomonadales;Veillonellaceae;Dialister*	0.4723	2.8018	4.3975	10.1129	0.0004	4-years
Escherichia coli	*Proteobacteria;Gammaproteobacteria;Enterobacteriales;Enterobacteriaceae;Escherichia/Shigella*	11.8918	14.1779	3.8256	10.3291	0.0010	1-year
Faecalibacterium	*Firmicutes;Clostridia;Clostridiales;Ruminococcaceae;Faecalibacterium*	2.4346	4.7598	9.3919	12.6895	0.0000	4-years

prausnitzii							
Fusicatenibacter saccharivorans	*Firmicutes;Clostridia;Clostridiales;Lachnospiraceae;Fusicatenibacter*	0.8787	1.6513	0.5529	1.0605	0.0425	1-year
Gemmiger formicilis	*Firmicutes;Clostridia;Clostridiales;Lachnospiraceae;Fusicatenibacter*	0.3705	1.6513	1.4567	1.0605	0.0038	4-years
Romboutsia timonensis	*Firmicutes;Clostridia;Clostridiales;Peptostreptococcaceae;Romboutsia*	1.5681	2.8120	0.3818	1.9485	0.0038	1-year
Roseburia faecis	*Firmicutes;Clostridia;Clostridiales;Lachnospiraceae;Roseburia*	0.2633	1.2861	1.9782	5.0183	0.0007	4-years
Roseburia inulinivorans	*Firmicutes;Clostridia;Clostridiales;Lachnospiraceae;Roseburia*	0.1667	0.6603	1.5116	5.6367	0.0037	4-years
Ruminococcus bromii	*Firmicutes;Clostridia;Clostridiales;Ruminococcaceae;Ruminococcus*	0.7746	2.1357	1.7286	5.1271	0.0200	4-years
Ruminococcus gnavus	*Firmicutes;Clostridia;Clostridiales;Lachnospiraceae;Mediterraneibacter*	4.5545	5.7721	2.1977	5.5324	0.0060	1-year
Streptococcus salivarius	*Firmicutes;Bacilli;Lactobacillales;Streptococcaceae;Streptococcus*	2.9009	5.1649	0.5903	1.0974	0.0024	1-year
Veillonella dispar	*Firmicutes;Negativicutes;Selenomonadales;Veillonellaceae;Veillonella*	3.6217	5.0458	0.8911	3.3873	0.0011	1-year
